# Simplifying multidimensional fermentation dataset analysis and visualization: One step closer to capturing high-quality mutant strains

**DOI:** 10.1038/srep39875

**Published:** 2017-01-03

**Authors:** Xiang Zhou, Dan Xu, Ting-Ting Jiang

**Affiliations:** 1 Institute of Modern Physics, Chinese Academy of Sciences, 509 Nanchang Rd., Lanzhou, Gansu, 730000, P.R.China; 2 University of Chinese Academy of Sciences, 19 A Yuquan Rd, Shijingshan District, Beijing, 100049, P.R.China

## Abstract

In this study, we analyzed mutants of *Clostridium acetobutylicum*, an organism used in a broad range of industrial processes related to biofuel production, to facilitate future studies of bioreactor and bioprocess design and scale-up, which are very important research projects for industrial microbiology applications. To accomplish this, we generated 329 mutant strains and applied principal component analysis (PCA) to fermentation data gathered from these strains to identify a core set of independent features for comparison. By doing so, we were able to explain the differences in the mutant strains’ fermentation expression states and simplify the analysis and visualization of the multidimensional datasets related to the strains. Our study has produced a high-efficiency PCA application based on a data analytics tool that is designed to visualize screening results and to support several hundred sets of data on fermentation interactions to assist researchers in more precisely screening and capturing high-quality mutant strains. More importantly, although this study focused on the use of PCA in microbial fermentation engineering, its results are broadly applicable.

Principal component analysis (PCA) is a statistical tool based on mathematical operations. It is widely used for high-dimensional data analysis, and it may be the most popular multivariate statistical technique used in almost all scientific disciplines. Its origin can be traced back to several mathematicians: Pearson^1^, Cauchy^2^, Jordan^3^, Cayley^4^, Hamilton^5^, and Boyer and Merzbach^6^. However, modern PCA was formalized by Hotelling^7^, who also suggested the ‘principal component’ element of its name, which was unprecedented in scientific terms. Within the fields of visualization and computer graphics alone, PCA has been used in applications including facial recognition^8^,^9^,^10^, motion analysis and synthesis^11^,^12^,^13^, clustering^14^,^15^,^16^, and dimension reduction^17^,^18^,^19^,^20^. PCA is a quantitatively rigorous method of data simplification^21^. Based on this method, a new set of variables called principal components^22^ is extracted, where each principal component is often a linear combination or a few or multiple of the original variables^23^. All of the principal components have the characteristic of being orthogonal to each other; thus, they contain no redundant information^24^. As a whole, the principal components of a set of data form an orthogonal basis for the data space^25^,^26^.

*Clostridium acetobutylicum (C. acetobutylicum*) is an industrially valuable bacterium that is sometimes identified by the term “Weizmann Organism”, named for the biochemist Chaim Weizmann. Weizmann’s nationality was Israeli-British and he was born in Russia. As a senior lecturer at the University of Manchester, England, he used this bacterium in 1916 as a biochemical tool to produce acetone, ethanol, and butanol from starch. This method has since been described as the acetone-butanol-ethanol fermentation process (ABE process); it yields acetone, butanol, and ethanol at a ratio of 3:6:1. Acetone was used in the important wartime task of casting cordite during the first world war and the second world war, and the alcohols were used to produce vehicle fuels and synthetic rubber^27^,^28^. Although ABE fermentation is one of the longest known large-scale biofermentation processes, the depletion of fossil fuels has renewed interest in ABE fermentation^29^,^30^. Between the 1890s and the 1990s, the number of research studies in this field increased considerably, with a focus on improving the overall process, including the development of alternative fermentation substrates, improved strains, improved cultivation techniques, and improved product-removal techniques^31^,^32^,^33^. During the 1990s and 2000s, intensive basic research studies investigated the genetics of solvent-producing Clostridium. sp. and strove to improve these strains through genetic manipulations^34^,^35^. Rapid scale-up trials were also performed at the beginning of the 21st century to improve traditional ABE fermentation methods. However, the economic feasibility of biobutanol production via ABE fermentation suffers from product toxicity, relatively low product yields with respect to the production bacteria, multiple end products, production inhibitions, and inefficient product recovery from the produced alcohol mixtures^36^,^37^,^38^.

Note that the productivity of metabolites can be improved by up to a factor of ten through suitable bacterium improvement techniques^39^. Mutagenesis is one of the most reliable and widely used approaches for strain improvement^40^. During organism breeding, mutations are induced using heavy-ion irradiation, ultraviolet (UV) rays, X-rays, γ-rays, lasers, neutrons, and thermophoresis^41^. Chemical physics methods based on methyl methane sulfonate (MMS), hydroxyl amine (HA), and N-methyl-N’-nitro-N-nitrosoguanidine (MNNG) have been adopted as important research methods for inducing mutations^42^. These mutagenesis approaches tend to produce abundant mutant strains. However, because of the problems posed by the need to handle the complex multidimensional data that describe the fermentation expression states of these mutant strains, researchers have made substantial effects to find ways to better screen and capture high-quality mutant strains. Fortunately, in these mutant strain expression datasets, which contain many variables, groups of variables often vary together. One reason for this behavior is that more than one variable might reflect the same driving principle governing the behavior of the system. In many systems, only a few such driving forces exist. However, abundant instrumentation enables the measurement of dozens of system variables. Therefore, researchers can take advantage of this redundancy of information and simplify the problem by replacing a group of variables with a single new variable.

In the previous scientific research literature dedicated to application of the approach in the analysis of datasets on fermentation processes. For example, the applicability of РСА for fermentation data analysis using eight fed-batch fermentations with a recombinant L-phenylalanine-producing Escherichia coli strain as a test system was investigated by Takors *et al*.^43^; experimental determination by principal component analysis of a reaction pathway of biohydrogen production by anaerobic fermentation was determined by Aceves-Lara *et al*.^44^; principal component analysis and partial least squares regression can be used to extract information from particle size distribution data and predict rheological properties was determined by Peterson *et al*.^45^; principal component analysis the measurement profiles acquired during the monitoring of several fed-batch fermentations for the production of erythromycin was applied by Bicciato *et al*.^46^. However, the number of РСА applications for fermentation dataset analysis and visualization are still rather limited, although this tool holds great promise, merit and interest.

A series of experiments involving the fermentation of mutant *C. acetobutylicum* have produced observations of the differential expressions of hundreds of mutant strains across multiple conditions. In this study, the application of PCA to these expression data enabled the direct comparison of a core set of independent features of the expression states of the 329 mutant strains that were investigated. Thus, we can explain the differences among the mutant strains’ multidimensional fermentation datasets and move one step closer to capturing high-quality mutant strains.

## Results and Discussion

### Source of the mutants’ fermentation multidimensional datasets

As shown in the Supplementary Information 1, the mutants all that were obtained, and they were from multiple rounds by the experiment of heavy-ion irradiation. 329 mutants were screened by MTT. It would be interesting to summarize how the mutants were generated, but this may be beyond the scope of this study since the focus of the manuscript is the PCA analysis. In addition, we are currently investigating and will dissect the top-performing mutants. All 329 mutants according to supplementary Information 1 (Materials & Methods for data procurement from mutant strains) conducted data acquisition. Table S1 (Supplementary Information 2) showed all multidimensional datasets that unlike otherwise noted, fermentation was carried out in serum bottles, this is the source of the datasets.

### Visualization of multidimensional datasets

**Figure 1:**
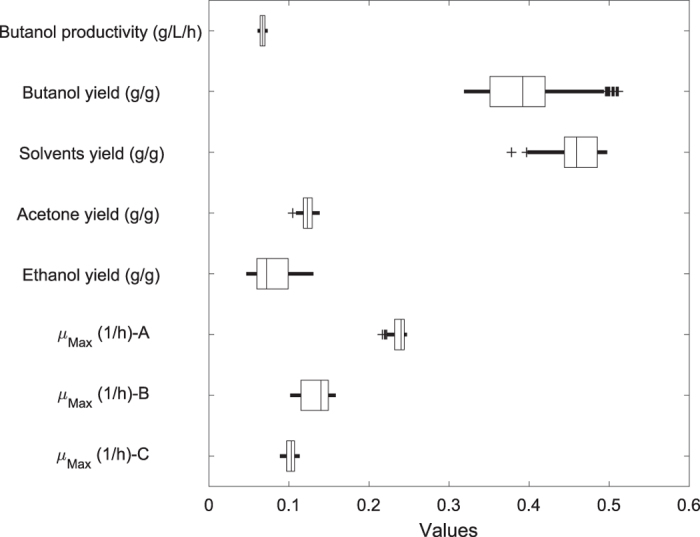
The distribution of the measured data matrix of 329 rows and 8 columns, corresponding to a dataset consisting of 329 mutant strains and 8 variables. These variables are butanol productivity (g/L/h), butanol yield (g/g), solvent (ABE) yield (g/g), acetone yield (g/g), ethanol yield (g/g), maximal specific growth rate (added butyrate: 5.0 g/L; *μ*_*Ma*x_-A), maximal specific growth rate (added butyrate: 6.5 g/L; *μ*_*Ma*x_-B), and maximal specific growth rate (added butyrate: 8.5 g/L; *μ*_*Ma*x_-C).

Table S1 (Supplementary Information 2) shows all of the effects of an increasing butyric acid concentration on the ABE fermentation yields from the substrate, the butanol productivity of fermentation for each of the 329 mutant strains supplemented with 5.0-g/L butyric acid, and the maximal specific growth rates of the 329 mutant strains. Hence, a dataset is obtained that consists of 329 mutant strains and 8 variables. The actual measurements can be arranged in a table or a matrix with dimensions of *329* × *8*. The variables are butanol productivity (g/L/h), butanol yield (g/g), solvent (ABE) yield (g/g), acetone yield (g/g), ethanol yield (g/g), maximal specific growth rate (added butyrate: 5.0 g/L; *μ*_*Max*_-A), maximal specific growth rate (added butyrate: 6.5 g/L; *μ*_*Max*_-B), and maximal specific growth rate (added butyrate: 8.5 g/L; *μ*_*Max*_-C). With 329 mutant strains and 8 columns (variables), obtaining an overview of the various types of information available in this multidimensional dataset is difficult. A good starting point is to plot the individual variables for the 329 mutant strains. As shown in Fig. 1, there is more variability in the values of the butanol and solvent (ABE) yields than in the values of butanol productivity and *μ*_*Max*_-C. Normally, one would consider plotting each of the original variables, but doing so would result in 32 variograms. Thus, the advantage of PCA is that it may reduce the number of variables that must be considered. Sometimes, the original data can be used to calculate the principal components, if the same unit is appropriate for each variable. However, when different variables in different columns have different units or when the variance in value among the columns is large, the data must be standardized to improve performance.

### Analysis of multidimensional datasets

The first three principal component coefficient vectors are shown in Table 1. As mentioned above, when all variables have the same unit, it is appropriate to compute the principal components from the raw data. The correlation matrix, which has dimensions of *8 × 8*, reveals that the variables are highly correlated (Table 1).

When the variables are expressed in different units, or if the variance in value among the columns is substantial, as in this study, scaling or weighting the data is preferred. The correlations between select variables can be as large as 0.33. PCA serves to construct new independent variables from linear combinations of the originals. For this purpose, in PCA, the inverse variances of the measured values are used as weights. The coefficient vectors for the first three principal components, namely, butanol productivity (g/L/h), butanol yield (g/g) and solvent (ABE) yield (g/g), are shown in Table 2. It is seen that the first principal component makes the largest contributions to the first and fifth variables: butanol productivity and ethanol yield. The coefficients of this principal component are positive. The principal component variables are defined as linear combinations of the original variables. The extracted eigenvector table provides the coefficients for the following equations:


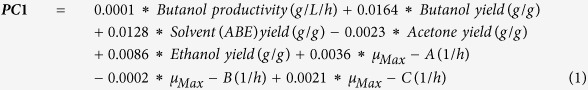



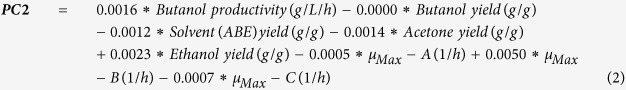



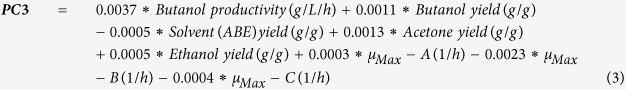


The coefficients for our data are weighted, and as a result, the coefficient matrix is not orthonormal. Thus, the coefficients were transformed to become orthonormal (Table 2). Based on the data presented in Table 2, this was accomplished using MATLAB code, with the following


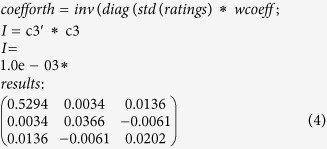


As shown above, the transformed coefficients are orthonormal.

### Interpreting the latent vector and visualizing the results

**Figure 2:**
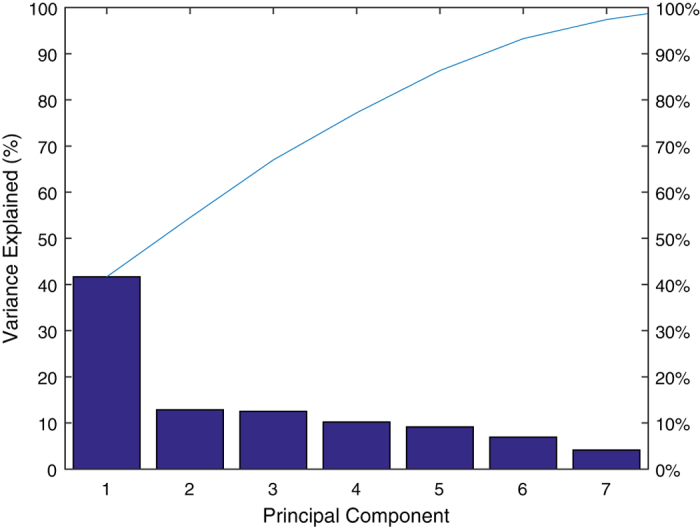
A scree plot of the percent variability explained by each principal component. This scree plot shows only the first seven (instead of all eight) components, which together explain 98% of the total variance. The only marked gap in the amount of variance accounted for by each component is between the first and second components. The first component alone explains less than 42% of the variance; thus, more components might be needed to adequately describe the data.

The latent vector describes the variability in the data that is explained by each principal component. Specifically, each independent column of the obtained score matrix has a variance equal to the value in the corresponding row of the latent vector. Based on the data presented in Tables S1, 1 and 2, using MATLAB, it is easy to calculate the elements of the latent vector: 3.3327, 1.0272, 0.9993, 0.8170, 0.7320, 0.5534, 0.3310, and 0.2074, respectively. The scree plot presented in Fig. 2 shows seven out of the eight components, which together account for 98% of the total variance. There is a large gap between the variances of the first and second components. Nevertheless, the first component alone accounts for less than 41.6582% of the variance, whereas the second component explains less than 12.8395% of the variance, and the third component explains less than 12.4917% of the variance. Consequently, multiple components might be needed to adequately describe the data. Figure 2 shows that the first three principal components together account for approximately 66% of the total variability in the standardized data values and thus may serve as a reasonable foundation for reducing the dimensionality of the data.

**Figure 3:**
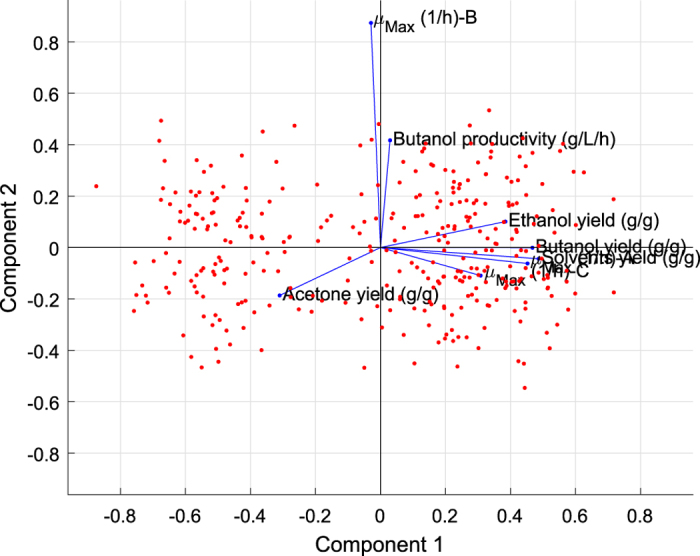
The 2D visualization, which includes one point for each of the 329 observations, with the coordinates indicating the scores of each observation for the two principal components represented in the plot. All eight variables are represented by vectors in this bi-plot, where the direction and length of each vector indicate how each variable contributes to the two principal components represented in the plot.

**Figure 4:**
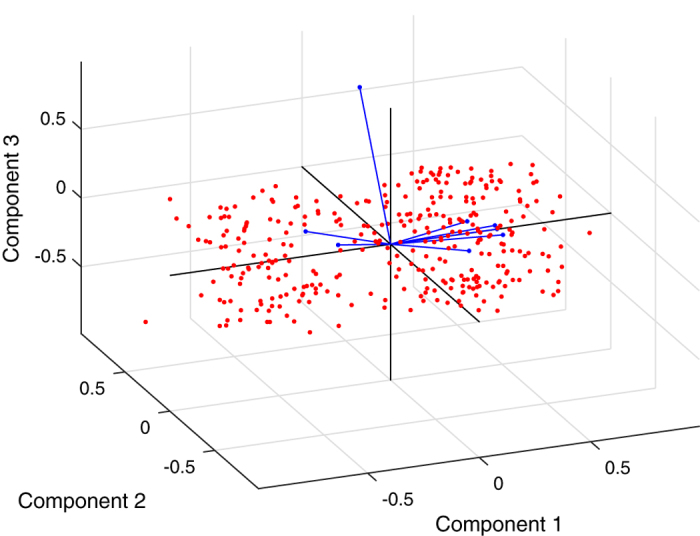
The 3D visualization, which includes one point for each of the 329 observations, with the coordinates indicating the scores of each observation for the three principal components represented in the plot. All eight variables are represented by vectors in this bi-plot, where the direction and length of each vector indicate how each variable contributes to the three principal components represented in the plot.

In Figs 3 and 4, all eight variables are plotted as vectors, where the direction and length of each vector indicate the contribution of the corresponding variable to each principal component. The first principal component, which is shown on the horizontal axis, has positive coefficients for six variables: butanol productivity (g/L/h), butanol yield (g/g), solvent (ABE) yield (g/g), ethanol yield (g/g), maximal specific growth rate (added butyrate: 5.0 g/L; *μ*_*Max*_-A), and maximal specific growth rate (added butyrate: 8.5 g/L; *μ*_*Max*_-C). Thus, the six corresponding vectors lie on the right side of the graph. The second and third elements of the coefficient vector for the first principal component, corresponding to the butanol yield and the solvent (ABE) yield, respectively, have the largest values (Fig. 3). The second principal component, which is shown on the vertical axis, has positive coefficients for the variables representing maximal specific growth rate (added butyrate: 6.5 g/L; *μ*_*Max*_-B), butanol yield (g/g), and ethanol yield (g/g) and negative coefficients for the remaining five variables (Fig. 3). Thus, the second component distinguishes between clusters of the 329 mutant strains that have high values for the first set of variables and low values for the second and clusters for which the opposite is true. Note that Fig. 4 is helpful for cases in which the first two principal components do not account for a sufficient amount of the variance in the multidimensional fermentation datasets. Here, the data points have been scaled with respect to the maximum score and the number of coefficients; therefore, only their relative locations can be found by using the graph.

### Capturing high-quality mutant strains

The multivariate distance of each observation from the center of a dataset can be measured using many methods; one common strategy is to use Hotelling’s *T*^*2*^ test, which was first introduced in 1931^47^. This is an analytical way to identify the most extreme points in a dataset^48^,^49^,^50^. Hotelling’s *T*^*2*^ test can be thought of as a supplement to the t-test; it can be applied to the scores obtained for a PCA model as follows:


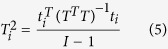


where the matrix of scores (*I* × *R*) obtained from the calibration samples is represented by *T* and *t*_*i*_ is the *R* × *1* vector representing the *R* scores for the *i*^*th*^ sample. Under the assumption that the scores are normally distributed, the confidence limits for *T*_*i*_^2^ can be assigned as follows:


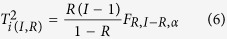


Thus, one of the highest-quality mutant strains can be quickly and accurately captured from the multidimensional fermentation datasets representing the 329 mutant strains. As mentioned earlier, this was accomplished using MATLAB code. Based on all previous data analyses, the most extreme point in the datasets was identified as follows:


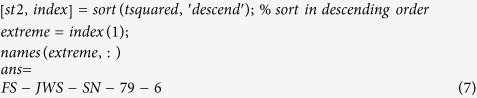


This extreme point is not surprising because the measured values for mutant FS-JWS-79-6 are the farthest from the average of the multidimensional fermentation datasets for the 329 mutant strains. In addition, mutants *FS-SXT-AP-R631, FS-SXT-LE-BH-A7, FS-SXT-GC-V9-77, FS-ZKS-TF-MT18* and *FS-ZKS -TF-ZT637* also appear to be more extreme than the other data.

### Influence of addition of butyric acid on extreme mutants

Most industrial processes are described to not use addition of external butyric acid as costs are prohibiting, e.g. the South African process^27^, the former Soviet Union process^51^ or the historic and current processes in China^52^. Yet, the presence of butyric acid in the fermentation broth has been shown to activate solvent production by *C. acetobutylicum*^53^,^54^,^55^. Additionally, increased yields and increased production of solvents have been reported following the addition of butyric and acetic acid to cultures of *Clostridium beijerinckii* and *C. acetobutylicum*^56^,^57^,^58^. The production of solvents, especially butanol, is clearly influenced by butyric acid. Adding butyric acid shifts the metabolic stage of the culture by decreasing the pH of the medium, and it can also be utilized by the cells as a co-substrate for the formation of butanol^59^,^60^,^61^,^62^,^63^. Therefore, controlling the amount of butyrate in the culture medium is of great industrial importance. However, butyric acid has previously been reported to inhibit cell growth^64^,^65^. The fermentation results presented in Table 3 show that cells of the mutant *FS-JWS-SN-79-6* showed a better “apparent” tolerance at butyric acid concentrations between 5.0 and 11.5 g/L, although this mutant did exhibit a gradual inhibition of cell growth; no growth was observed for butyric acid concentrations above 11.5 g/L, and the effect of increasing the butyric acid concentration on the batch efficiency parameters (yield from the substrate and productivity) was investigated for this mutant. Often times strains that have a high tolerance (to butyric acid) do not have the highest production rates (of butanol). It is very interesting that the wild-type *C. acetobutylicum ATCC 824* showed (low) butanol production levels in contrast to extreme mutants *FS-JWS-SN-79-6* which produced under absence of butyrate supplementation. The results presented in Table 4 shows the mutant *FS-JWS-SN-79-6* that produces more butanol without supplemented butyrate (0.262 g/g) than the wild-type strain. The cells unaffected by butyric acid addition consume *Clostridium* growth medium (CGM) mainly for growth producing simultaneously butyrate as primary metabolite. As butyrate builds up in the system the medium pH drops with the cells shifting their metabolic state from acidogenesis to solventogenesis. It is obvious that extreme mutants *FS-JWS-SN-79-6* have strong ability of metabolic. This resulted in a 2.2-fold increase in butanol yield from substrate coupled with 3.1-times more butanol productivity. In addition, the maximal specific growth rates results presented in Table 3 show that butyric acid (5.0 g/L) was slightly added in the media before the optimal point was reached, solvents production was stimulated at the cost of lower specific cell growth rates but with moderate biomass levels. The outcome was a significant increase in the solvents yields from biomass for all strains. The fact that these bacteria were metabolically inactive for solvent production due to low butyric acid levels and suboptimal pHs necessary for solvents production, reflected into high cell growth rates with resultant high biomass levels in the system. As showing in Table 3, the results demonstrate that butyric acid has effectively a prominent inhibitory effect on cell growth with all specific growth rates declining with increasing butyrate concentrations. This finding confirms previous results obtained with *Clostridium butyricum* grown in a glucose-limited chemostat culture^66^. While cells from *C. beijerinckii* BA 101 could be considered the most resistant ones to critical concentrations of butyrate (10 g/L), cells of *C. beijerinckii* ATCC 55025 evidenced a better “apparent” tolerance in the butyric acid region between 2~8 g/L. In all cases one can see that butyrate feeding favors ABE-solvents production over the control cultures. Above the optimal butyrate feeding concentration (5.0 g/L) cell growth is strongly inhibited lessening butanol yields and productivities for all strains. As showing in Tables 3 and 4, in contrast, as the butanol production pathway of extreme mutants *FS-JWS-SN-79-6* is induced by the addition of external butyric acid (5.0 g/L), the levels of *butyryl-CoA* are increased from *acetoacetyl-CoA* instead of forming acetoacetate. This results in a lower acetone production. This corresponded to a 1.1-fold discrease in the acetone yield from 0.126 g/g to 0.113 g/g. Likewise, the wild-type *C. acetobutylicum ATCC 824* showed (low) acetone yield levels which produced under butyrate supplementation (5.0 g/L). Further details on the metabolic pathways for butanol and acid production can be found elsewhere^34^,^67^.

### Evaluation of the mutants from the ABE fermentation perspective

In all cases, 5.0 g/L was found to be the optimal concentration of butyric acid for maximizing the yield for all ABE solvents and the butanol productivity. Adding butyric acid to the medium significantly increased the production of butanol, resulting in a global maximum productivity of 0.068 g/L/h in the fermentation broth for this mutant. This corresponded to a 2.28-fold increase in the butanol yield from the substrate (0.183 g/g), coupled with a 3.09-fold increase in productivity (0.022 g/L/h) (Table 3). Moreover, high intracellular concentrations of this acid activated the enzymes to produce neutral products. Thus, the mutant *FS-JWS-SN-79-6* will synthesize the enzymes for butanol production at pH 7 as the butyrate concentration in the medium increases. In similar experiments, cells have been routinely observed to continue to grow when supplemented with butyric acid at concentrations of 5.0~8.5 g/L and, in some cases, above 11.5 g/L. Above the optimal level of butyric acid concentration, the yield values decrease as a direct consequence of gradual cell growth inhibition by the co-substrate with concomitant low biomass concentration coupled by low levels of butanol produced. ABE formation of ethanol and acetone limits the amount of metabolic precursors available for butanol production^68^. As showing in Tables 3 and 4, the results demonstrate that acetone and ethanol production levels were not significantly affected. Based on supplementation with 5.0-g/L butyric acid compared to the wild-type strain, the conversion of CGM to butanol yield by the mutant *FS-JWS-SN-79-6* had increased with 43.8%, and total ABE solvent yields from CGM were up with 47.6%. In absence of butyrate supplementation compared to the wild-type strain, the conversion of CGM to butanol yield by the mutant *FS-JWS-SN-79-6* had increased with a 1.6-fold, and total ABE solvent yields from CGM were up with a 1.3-fold. Above the results correspond to the previous investigations, to restore solvent productivity, Nair and Papoutsakis (1994) expressed the alcohol dehydrogenase gene (*adhE*), normally located on *pSOL1*, in strain M5 from a replicative plasmid^69^. Butanol yield was restored without acetone formation, but at reduced levels compared to the wild-type strain, while large amounts of acetate and butyrate accumulated in the growth medium.

High-production of biobutanol by *Clostridium* have been reported detailedly in the following research literature. Such as hyper-butanol producing strains from various mutagenesis strategy and mutants, including acetone-butanol-ethanol production with high productivity using *Clostridium acetobutylicum* BKM19^70^, acetone-butanol-ethanol production from cane molasses using *Clostridium beijerinckii* mutant obtained by combined low-energy ion beam implantation and N-methyl-N-nitro-N-nitrosoguanidine induction^71^, comparative genomic and transcriptomic analysis revealed genetic characteristics related to solvent formation and xylose utilization in *Clostridium acetobutylicum* EA 2018^72^ and recent advances in ABE fermentation: hyper-butanol producing *Clostridium beijerinckii* BA101^73^. Yields from biomass up to 17.6 g/L of butanol and the maximum butanol and ABE productivities of 9.6 and 20.0 g/L/h from 85.2 g/L glucose in the non-mutant *C. acetobutylicum* batch fermentation were obtained^70^. Supplementing the fermentation medium (MP2) with sodium acetate enhances solvent production to 33 g/L by in the non-mutant *Clostridium beijerinckii* BA101^73^. The production of butanol was 15.8 ± 0.7 g/L by *Clostridium beijerinckii* L175 after mutagenesis technique with N^+^ ion implantation^71^. In contrast, our results presented in Table 4 show that mutant *FS-JWS-SN-79-6* was also enhanced to improve butanol production from 7.73 to 18.43 g/L after ^12^C^6+^ heavy ion irradiation. Clearly, Mutant *FS-JWS-SN-79-6* produces more butanol without supplemented butyrate (18.43 g/L) than the *C. acetobutylicum* ATCC 824. Our findings suggested using ^12^C^6+^ heavy ion irradiation favors ABE-solvents production over the non-radiated strain and other mutagenesis strategy. The strategy reported here may contribute to develop a cost-effective butanol fermentation process, making it competitive compared with similar fermentation processes.

## Conclusion

PCA is a multivariate method that is used to examine datasets in which the observations can be expressed in terms of many inter-correlated quantitative dependent variables. Furthermore, PCA can be simplified to a correspondence analysis that handles qualitative variables. It can also be regarded as a multiple factor analysis that handles heterogeneous sets of variables. The purpose of PCA is to determine relevant information from a dataset, characterize it in terms of a set of new orthogonal variables (principal components), and visualize the patterns of similarity in the variables and observations as specific locations on a map. In this work, data on a large number of mutant strains of *C. acetobutylicum* secreted after mutagenesis were collected. For industrial ABE fermentation, distinguishing between high-quality and mediocre producing mutants is highly important. Because these organisms’ multidimensional fermentation datasets contain many correlated variables, PCA can serve as an inexpensive, efficient and reliable approach for identifying high-quality mutants. Through data acquisition, normalization, analysis simplification, and visualization, the proposed interactive approach helps users to understand and rapidly apply PCA by creating a visual model in their minds. Finally, the most extreme points are clearly evident, allowing high-quality mutant strains to be easily captured. In conclusion, the information obtained in this research will support further studies of bioreactor and bioprocess design and scale-up, which are very important topics for ABE industrial applications.

## Methods

### Cultures and medium

To test the production of butanol by various strains, a rich P2 medium containing 60 g/L glucose, 3.6 g/L yeast extract, 2.7 g/L peptone, 3.2 g L K_2_HPO_4_, 3.2 g/L KH_2_PO_4_, 0.2 g/L MgSO_4_, 0.2 g/L MnSO_4_, 0.02 g/L FeSO_4_, 0.02 g/L NaCl, 1.5 g/L yeast extract (Difco, USA), 2.5 g/L ammonium acetate, 0.0005 g/L p-aminobenzoate, 0.0005 g/L thiamin, 0.00005 g/L biotin, and 35 μg/mL thiamphenicol was used. To test the production of butanol from different substrates, the same rich P2 medium with 30 g/L instead of 60 g/L glucose was used^73^,^74^. Unless otherwise noted, the fermentation was conducted in serum bottles, each of which contained 40 mL of the medium and was inoculated with 1% (v/v) of an overnight culture in Reinforced *Clostridial* Medium (RCM; Difco, Detroit, MI, USA) at 37 °C and 250 rpm. The pH was maintained between 5.0 and 6.5 by adding NaOH solution twice a day^75^.

### Microorganisms and breeding

*Clostridium acetobutylicum* ATCC 824 was obtained from the Drug R & D Center of Institute of Modern Physics, Chinese Academy of Sciences, China. All bacteria were maintained in P2-medium at 4 °C as stock cultures. To prepare inocula of all 4-strains in totally anoxic conditions the following procedure was employed: serum tubes containing 5.0 ml of P2-medium were first purged with sterile nitrogen gas for 5-min. To prevent caramelization of sugar, a browning reaction, a separate 50 g⋅l-1 dextrose solution in distilled water was prepared in a 100-mL serum bottle and purged with nitrogen gas for 15-min again to attain perfect anaerobic conditions^76^,^77^. Both vessels were tightly sealed with rubber stoppers and aluminum crimps to prevent ingress of air and contamination with oxygen. Both liquids were sterilized by autoclaving at 121 °C, 15 psig for 20-min after which they were left at room temperature for cooling. 1.0 ml of dextrose solution was then added to the first tube followed by cell inoculation with 1/30 volume of each original stock culture. Anaerobic stock cultures for all strains were taken from an original serum tubes stored at 4 °C. Prior to inoculation the stock culture tubes were left resting at room temperature for 30-min inorder to pre-activate the cells. The pre-culture was incubated at 37 °C during 16-hours for cell growth followed by another inoculation around in order to obtain final fresh cell culture inocula.

### Experimental setup and heavy-ion beam irradiation

The experiment was performed at the Cancer Therapy Terminal of the Heavy Ion Research Facility at Lanzhou (HIRFL). The upgraded accelerator system of HIRFL consists of a Sector Focus Cyclotron (SFC), a Separated Sector Cyclotron (SSC), the main Cooling Storage Ring (CSRm), and the experimental Cooling Storage Ring (CSRe). High-energy ^12^C^6+^-ions with an energy of 196 AMeV were extracted by CSRm. Energies of 117 AMeV was obtained by adding the absorbers (water) and calibrating using the LISE program, and the corresponding uncertainty of the energies is not higher than 0.27%^78^. The extraction time of the carbon ions (approximately 10^6^–10^8^ ions/pulse) was approximately 3 s, and the priming dose was 80 Gy. The dose rates were up to 10 Gy/min. The temperature of the ^12^C^6+^ heavy-ion beams was <35 °C under these conditions^79^. For irradiation experiments, strains cells were grown in microcentrifuge tube (5 mL) to reach 90% confluence and they were completely filled with Dulbecco’s modified Eagle’s medium to avoid artifacts by irradiation through air layers.

### Totally anoxic conditions

Serum tubes containing 7.0 mL of P2 medium were first purged with sterile 80% N_2_, 10% CO_2_, and 10% H2 for 9 min. To prevent caramelization of the sugar, which is a browning reaction, a separate 60 g/L dextrose solution in distilled water was prepared in a 120-mL serum bottle and purged with nitrogen gas for 18 min to attain completely anaerobic conditions. Both vessels were tightly sealed with rubber stoppers and aluminum crimps to prevent the ingress of air and contamination with oxygen. Both liquids were sterilized by autoclaving at 121 °C and 15 psig for 25-min and were then cooled at room temperature. After 1.5 mL of dextrose solution was added to the first tube, the tube was inoculated with a 0.04 volume of each original stock culture. The anaerobic stock cultures of all of the strains were collected from the original serum tubes stored at 4 °C. Prior to inoculation, the stock culture tubes were incubated at room temperature for 25 min to pre-activate the cells. The pre-culture was incubated at 37 °C for 36 h to allow cell growth and then inoculated to obtain the final fresh cell culture inocula^80^.

### The source of the multidimensional fermentation dataset

The generation of mutant strains, the fermentation screening, the measurements and analytical methods accompanies this paper at Supplementary Information 1.

### Simplifying multidimensional fermentation dataset analysis and visualization

PCA is performed by determining the eigenvalues and eigenvectors of a covariance matrix. This covariance matrix is then utilized to determine the variation in the values of each dimension with respect to the mean. The dimensions of the data considered in our study can be described as random variables and often vary together. Such a relationship can be described as follows:


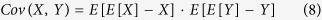


where *E[X]* and *E[Y]* are the expected values of *X* and *Y*, respectively. This can be further written as follows for a sampled dataset:


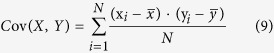


where 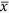 and 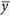 are the mean values of *X* and *Y*, respectively, and *N* is the number of dimensions of the dataset. The covariance matrix is then defined as *A*_*i,*j_ = *Cov (i, j*), where the data have been mean centered.

For an element of the covariance matrix, the sign is more important than the magnitude. For example, if the sign is positive, it indicates that both of the corresponding dimensions (*X* and *Y*) increase simultaneously. Conversely, if the sign of a matrix element is negative, it indicates that when one of the corresponding dimensions increases, the other decreases. When the covariance is zero, the two dimensions are independent of each other. According to the commutative property, *Cov (X, Y*) =* Cov (Y, X)*.

The eigenvalues and eigenvectors of interest are computed using the covariance matrix. Then, the eigenvalues are arranged in descending order, creating an order of significance. The eigenvector with the largest eigenvalue is considered to be the most dominant principle component (*PC1*), which describes the most significant relationship. The principal components are calculated through multiplication of the eigenvectors by the stratified eigenvalues.

PCA can be used as a dimension-reduction method through determination of the principal components of the input data. However, for the transformation of a high-dimensional dataset into a lower-dimensional space, the ideal low-dimensional space must be found from the eigenvectors of the covariance matrix. The ideal low-dimensional space minimizes the error between the input dataset and the PCA results based on


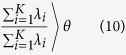


where *K* is the number of dimensions chosen from among the original *N* dimensions of the matrix, *θ* is the threshold criterion (typically 0.9 or 0.95), and λ is an eigenvalue. Using this information, the N × N matrix is linearly transformed into an *N* × *K* matrix. Although the number of dimensions decreases with the application of PCA, the difference between the input and output matrices is small. Common values of *K* are 2 and 3, which correspond to the mapping of a dataset into 2D and 3D coordinate systems, respectively.

## Additional Information

**How to cite this article**: Zhou, X. *et al*. Simplifying multidimensional fermentation dataset analysis and visualization: One step closer to capturing high-quality mutant strains. *Sci. Rep.* **7**, 39875; doi: 10.1038/srep39875 (2017).

**Publisher's note:** Springer Nature remains neutral with regard to jurisdictional claims in published maps and institutional affiliations.

## Supplementary Material

Supplementary Information 1

Supplementary Information 2

## Figures and Tables

**Table 1:** In the correlation matrix, which has a size of 8 × 8, the variables are highly correlated, with many having correlation values exceeding 0.45.

	Butanol productivity (g/L/h)	Butanol yield (g/g)	Solvents (ABE) yield (g/g)	Acetone yield (g/g)	Ethanol yield (g/g)	*μ*_*Max*_-A (1/h)	*μ*_*Max*_-B (1/h)	*μ*_*Max*_-C (1/h)
Butanol productivity (g/L/h)	**1.000000**	0.062521	0.016778	−0.002446	0.047993	0.027159	0.009812	6.699713e−04
Butanol yield (g/g)	0.062521	**1.000000**	0.781528	−0.386327	0.444585	0.643167	−0.027757	0.409016
Solvents (ABE) yield (g/g)	0.016778	0.781528	**1.000000**	−0.422182	0.513211	0.654756	−0.053534	0.463406
Acetone yield (g/g)	−0.002446	−0.386327	−0.422182	**1.000000**	−0.308724	−0.349048	−0.040566	−0.202345
Ethanol yield (g/g)	0.047993	0.444585	0.513211	−0.308724	**1.000000**	0.588894	0.012975	0.245488
*μ*_*Max*_-A (1/h)	0.027159	0.643167	0.654756	−0.349048	0.588894	**1.000000**	−0.084181	0.318853
*μ*_*Max*_-B (1/h)	0.009812	−0.027757	−0.053534	−0.040566	0.012975	−0.084181	**1.000000**	−0.029020
*μ*_*Max*_-C (1/h)	6.699713e−04	0.409016	0.463406	−0.202345	0.245488	0.318853	−0.029020	**1.000000**

*All experiments were repeated 3 times, and the average values are reported.

**Table 2:** The principal component variables are defined as linear combinations of the original variables.

	*Coefficients of PC1*	*Coefficients of PC2*	*Coefficients of PC3*
Butanol productivity (g/L/h)	0.0001	0.0016	0.0034
Butanol yield (g/g)	0.0164	0.0000	0.0011
Solvents (ABE) yield (g/g)	0.0128	−0.0012	−0.0005
Acetone yield (g/g)	−0.0023	−0.0014	0.0013
Ethanol yield (g/g)	0.0086	0.0023	0.0005
μ_Max_-A (1/h)	0.0036	−0.0005	0.0003
μ_Max_-B (1/h)	−0.0002	0.0050	−0.0023
μ_Max_-C (1/h)	0.0021	−0.0007	−0.0004

The table of the extracted eigenvectors provides the coefficients for the equations.

**Table 3:** Based on supplementation with 5.0-g/L butyric acid, the effects of increasing the butyric acid concentration (6.5, 8.5 and 11.5 g/L) on the ABE fermentation parameters from the substrate, the butanol productivity, and the maximal specific growth rates for *C. acetobutylicum* ATCC 824 and mutant strains were determined as shown below.

Bacterium	Butanol productivity (g/L/h)	Butanol yield (g/g)	Solvents (ABE) yield (g/g)	Acetone yield (g/g)	Ethanol yield (g/g)	Maximal specific growth rate (added butyrate 5.0 g/L) *μ*_*Max*_ (1/h)	Maximal specific growth rate (added butyrate 6.5 g/L) *μ*_*Max*_ (1/h)	Maximal specific growth rate (added butyrate 8.5 g/L) *μ*_*Max*_ (1/h)	Maximal specific growth rate (added butyrate 11.5 g/L) *μ*_*Max*_ (1/h)
*C. acetobutylicum ATCC 824*	0.022	0.183	0.235	0.076	0.024	0.183	0.087	0.069	—
Mutant *FS-JWS-SN-79-6*	0.068	0.418	0.494	0.113	0.060	0.224	0.146	0.093	0.057
Mutant *FS-SXT-AP-R631*	0.070	0.401	0.528	0.117	0.126	0.247	0.159	0.108	0.032
Mutant *FS-SXT-LE-BH-A7*	0.073	0.405	0.53	0.118	0.128	0.240	0.157	0.111	0.041
Mutant *FS-SXT-GC-V9-77*	0.068	0.435	0.533	0.113	0.117	0.243	0.157	0.110	0.029
Mutant *FS-ZKS-TF-MT18*	0.072	0.435	0.524	0.113	0.091	0.241	0.152	0.113	0.036
Mutant *FS-ZKS -TF-ZT637*	0.067	0.426	0.532	0.109	0.101	0.245	0.147	0.112	0.038

*All the experiments were repeated 3 times and the average value was taken.

**Table 4:** The performance of the best mutants against the wild-type in absence of butyrate supplementation on the ABE fermentation parameters from the substrate, the butanol productivity, and the maximal specific growth rates for *C. acetobutylicum* ATCC 824 and mutant *FS-JWS-SN-79-6* were determined as shown below.

Bacterium	Butanol Production (g/L)	Butanol productivity (g/L/h)	Butanol yield (g/g)	Solvents (ABE) yield (g/g)	Acetone yield (g/g)	Ethanol yield (g/g)	Maximal specific growth rate *μ*_*Max*_ (1/h)
*C. acetobutylicum ATCC 824*	7.73	0.017	0.164	0.318	0.083	0.031	0.236
Mutant *FS-JWS-SN-79-6*	18.43	0.048	0.262	0.413	0.126	0.068	0.242
