# Supplementary material for: Simplifying multidimensional fermentation dataset analysis and visualization: One step closer to capturing high-quality mutant strains
Source: Sci Rep. 2017 Jan 3;7:39875. doi: 10.1038/srep39875 (PMC5206668; doi:10.1038/srep39875)
Supplement: Supplementary Information 1 [file srep39875-s1.pdf]

Supplementary Information

Simplifying multidimensional fermentation dataset analysis and visualization: One step closer to capturing high-quality mutant strains

Xiang Zhou[1, †, *], Dan Xu[1, †] and Ting-Ting Jiang[1,2,†]

Author affiliations

[1] Institute of Modern Physics, Chinese Academy of Sciences, 509 Nanchang Rd., Lanzhou, Gansu, P.R.China 730000

[2] University of Chinese Academy of Sciences, 19 A Yuquan Rd, Shijingshan District, Beijing, P.R.China 100049

†**These authors contributed equally to this work.**

Corresponding Author:

Xiang Zhou

***Corresponding Email address:** syannovich@gmail.com or syannovich@impcas.ac.cn

Phone: 86-931-4969688 Fax: +86-931-4969698

Supplementary Information: 7 pages

Materials & Methods for data procurement from mutant strains

Cultures and medium

To test the production of butanol by various strains, a rich P2 medium containing 60 g L^{-1} glucose, 3.6 g L^{-1} yeast extract, 2.7 g L^{-1} peptone, 3.2 g L^{-1} K_2HPO_4, 3.2 g L^{-1} KH_2PO_4, 0.2 g L^{-1} $MgSO_4$, 0.2 g L^{-1} $MnSO_4$, 0.02 g L^{-1} $FeSO_4$, 0.02 g L^{-1} NaCl, 1.5 g L^{-1} yeast extract (Difco, USA), 2.5 g L^{-1} ammonium acetate, 0.0005 g L^{-1} p-aminobenzoate, 0.0005 g L^{-1} thiamin, 0.00005 g L^{-1} biotin, and 35 μg mL^{-1} thiamphenicol was used[1,2]. To test the production of butanol from different substrates, the same rich P2 medium with 30 g L^{-1} instead of 60 g L^{-1} glucose was used. Unless otherwise noted, the fermentation was conducted in serum bottles, each of which contained 40 mL of the medium and was inoculated with 1% (v/v) of an overnight culture in Reinforced Clostridial Medium (RCM; Difco, Detroit, MI, USA) at 37°C and 250 rpm. The pH was maintained between 5.0 and 6.5 by adding NaOH solution twice a day[3].

Microorganisms and breeding

Clostridium acetobutylicum ATCC 824 was obtained from the Drug R & D Center of Institute of Modern Physics, Chinese Academy of Sciences, China. All bacteria were maintained in P2-medium at 4°C as stock cultures. To prepare inocula of all 4-strains in totally anoxic conditions the following procedure was employed: serum tubes containing 5.0 ml of P2-medium were first purged with sterile nitrogen gas for 5-min. To prevent caramelization of sugar, a browning reaction, a separate 50 g·l^{-1} dextrose solution in distilled water was prepared in a 100 ml serum bottle and purged with nitrogen gas for 15-min again to attain perfect anaerobic conditions[4,5]. Both vessels were tightly sealed with rubber stoppers and aluminum crimps to prevent ingress of air and contamination with oxygen. Both liquids were sterilized by autoclaving at 121°C, 15 psig for 20-min after which they were left at room temperature for cooling. 1.0 ml of dextrose solution was then added to the first tube followed by cell inoculation with 1/30 volume of each original stock culture. Anaerobic stock cultures for all strains were taken from an original serum tubes stored at 4 °C. Prior to inoculation the stock culture tubes were left resting at room temperature for 30-min inorder to pre-activate the cells. The pre-culture was incubated at 37°C during 16-hours for cell growth followed by another inoculation round in order to obtain final fresh cell culture inocula.

Experimental setup and heavy-ion beam irradiation

The experiment was performed at the Cancer Therapy Terminal of the Heavy Ion Research Facility at Lanzhou (HIRFL). The upgraded accelerator system of HIRFL consists of a Sector Focus Cyclotron (SFC), a Separated Sector Cyclotron (SSC), the main Cooling Storage Ring (CSRm), and the experimental Cooling Storage Ring (CSRe). High-energy $^{12}C^{6+}$-ions with an energy of 196 AMeV were extracted by CSRm. Energies of 117 AMeV was obtained by adding the absorbers (water) and calibrating using the LISE program, and the corresponding uncertainty of the energies is not higher than 0.27%[6]. The extraction time of the carbon ions (approximately 10^6--10^8 ions/pulse) was approximately 3 s, and the priming dose was 80 Gy. The dose rates were up to 10 Gy/min[7]. The temperature of the $^{12}C^{6+}$ heavy-ion beams was <35°C under these conditions. For irradiation experiments, strains cells were grown in microcentrifuge tube (5 mL) to reach 90% confluence and they were completely filled with Dulbecco's modified Eagle's medium to avoid artifacts by irradiation through air layers.

MTT assay

The protocol is adapted from literature methods [8,9]. Briefly, for the irradiation experiments, the strain cells were grown in flasks (12 cm^2) to reach 90% confluence, and the flasks were then completely filled with DMEM to avoid artifacts due to irradiation through air layers. The protocol was adapted from methods described in the literature. Briefly, the DMEM in each well was supplemented with 100 μL of MTT (3-(4,5-dimethylthiazol-2-yl)-2,5-diphenyltetrazolium bromide) reagent (c=0.5 g L^{-1}), and the plate was incubated for 30 min at 37°C. The MTT solution was then removed. After the addition of 180 μL of DMSO, the plates were incubated for 15 min at 37°C to dissolve the formazan crystals. The absorbances of the DMSO extracts were determined at 560 nm with a reference wavelength of 690 nm using a Tecan Infinite F 200 microplate reader (Crailsheim, Germany). The treated cells were harvested the next day by trypsinization and counted, and a specific number of cells (900 and 450 strain cells) were plated in Petri dishes in triplicate for the clonogenic assay. Multiple MTT assays were performed using 96-well-plates with 3,000 and 6,000 cells per well, respectively. The survival fraction was calculated from the following equation:

$$Survival = 2^{-\frac{t_{delay}}{t_{doublingtime}}} \quad (1)$$

where the doubling time is the period of time required for a quantity of cells to double, and t_{delay} is the period of time required to reach a specific absorption value of the control versus irradiated strains cell.

Totally anoxic conditions

Serum tubes containing 7.0 mL of P2 medium were first purged with sterile 80% N_2, 10% CO_2, and 10% H_2 for 9 min. To prevent caramelization of the sugar, which is a browning reaction, a separate 60 g L^{-1} dextrose solution in distilled water was prepared in a 120-mL serum bottle and purged with nitrogen gas for 18 min to attain completely anaerobic conditions. Both vessels were tightly sealed with rubber stoppers and aluminum crimps to prevent the ingress of air and contamination with oxygen[10]. Both liquids were sterilized by autoclaving at 121°C and 15 psig for 25-min and were then cooled at room temperature. After 1.5 mL of dextrose solution was added to the first tube, the tube was inoculated with a 0.04 volume of each original stock culture. The anaerobic stock cultures of all of the strains were collected from the original serum tubes stored at 4°C. Prior to inoculation, the stock culture tubes were incubated at room temperature for 25 min to pre-activate the cells. The pre-culture was incubated at 37°C for 36 h to allow cell growth and then inoculated to obtain the final fresh cell culture inocula[11].

Biomass concentration estimation

Biomass concentration for each bacterial strain was estimated by dry cell weight (DCW) measurements using a predetermined correlation curve obtained between the absorbance measured at 600 nm and the cell dry weight (g·l-1). One unit of OD600 was roughly equivalent to 0.71 g·l^{-1} of DCW for cells of mutant strains grown in P2-medium.

The maximal specific growth rate and yields from substrate and biomass

The maximal specific growth rate (μ_{Max}) was determined from the semi-logarithmic plot described by equation (2,3) for data taken exclusively in the exponential phase of cell growth using a minimum requirement of three experimental data points[12,13]:

$$\ln OD_t = \mu_{\max} \cdot t + \ln OD_i \qquad (2)$$

$$\mu_{Max} = \Delta lnOD / \Delta t = \frac{lnOD(OD_t / OD_i)}{t - t_i} \qquad (3)$$

The turbidity parameter, OD, represents the optical density (absorbance) of the cell suspension measured at 600 nm of wavelength, and t is the sampling time (hours).

To estimate the overall solvent yields based on glucose plus butyric acid utilizations ($Y_{P/S}$) and biomass produced ($Y_{P/X}$), the two following equations were utilized:

$$Y_{P/S} = P/S_c \quad and \quad Y_{P/X} = P/X_{\max} \qquad (4)$$

Where P is the solvent production (g · l^{-1}), S_c is the substrate consumed, and X_{max} is the maximum biomass concentration attained during cell growth (g · l^{-1}).

Fermentation kinetics study

One hundred and fifty milliliters of the strain cell suspension prepared in serum bottles was inoculated into the fermentor, and the cells were then allowed to grow for 65-72 h at 37°C under agitation at 170 rpm and a pH value in the range of 6.0 to 6.3, which was controlled by the addition of OH^{-1}. After approximately 48 h of continuous circulation, most of the strain cells were immobilized, and no change in the cell density in the medium could be identified. For some experiments, P2 medium was used instead of RCM. The spent medium in the fermentor was then replaced with fresh medium, and the recirculation rate was increased to 200 mL min^{-1} for the new batch fermentation. For the main fermentation, *Clostridium* growth medium (CGM), which contains 35-100 g of glucose, 0.56 g of K_2HPO_4, 0.63 g of $MgSO_4{\cdot}7H_2O$, 0.015 g of $MnSO_4{\cdot}7H_2O$, 0.015 g of $FeSO_4{\cdot}7H_2O$, 1.7 g of NaCl, 2.1 g of asparagines, 6.5 g of yeast extract, and 1.35 g of $(NH_4)_2SO_4$ in 1.25 L of distilled water, was used. A modified reinforced *Clostridium* medium (RCM, Difco), which contains the following ingredients per liter of distilled water, was used for the pre-culture and solid culture an: 15 g of glucose, 12 g of tryptose, 10 g of beef extract, 3.6 g of yeast extract, 4.5 g of NaCl, 1.3 g of soluble starch, 0.65 g of cysteine hydrochloride, and 2.5 g of sodium acetate in 15 g L^{-1} agar. After the strains was pre-cultured using RCM at 37°C and pH 6.0-6.2 under anaerobic conditions, the grown cells were inoculated (15%, v/v) into CGM containing glucose. The main fermentation was conducted in a 5-L stirred-tank automatically assume chemostat fermentor (Marubishi MD-300) with a working volume of 1.5 L, and the pH was controlled at pH 6.0 using 2.5 M NaOH[14].

Analytical methods

A high-performance liquid chromatography (HPLC) system was used to analyze the carbohydrate compounds, including glucose, in the fermentation broth. The HPLC system consisted of an automatic injector (Agilent 1100, G1313A), a pump (Agilent 1100, G1311A), a Zorbax carbohydrate analysis column (250 mm×4.6 mm,

5 μm; Agilent, USA), a column oven maintained at 30°C (Agilent 1100, G1316A), and a refractive index detector (Agilent 1100, G1362A). The mobile phase was ethyl nitrile (ethyl nitrile/water=75:25) at a flow rate of 1.5 mL min^{-1}. Butyric acid and acetic acid were analyzed with a GC-2014 Shimadzu gas chromatograph (GC) (Shimadzu, Columbia, MD, USA) equipped with a flame ionization detector and a 30.0-m fused silica column (0.25-mm film thickness and 0.25-mm ID, Stabilwax-DA). The GC was operated at an injection temperature of 200°C, and 1 μL of sample was injected using the AOC-20i Shimadzu auto injector. The column temperature was maintained at 80°C for 3 min, raised to 150°C at a rate of 30°C min^{-1}, and maintained at 150°C for 3.7 min. The cell density was analyzed by measuring the optical density (OD) of the cell suspension at 600 nm using a spectrophotometer (Thermo Spectronics, Genesys 20, USA) with a conversion of 0.78 g L^{-1} of dry cell weight (DCW) per OD unit. The dry weight of the immobilized cell biomass was determined by centrifuging the fermentation broth at 10,000 g for 10 min, washing the sediment with distilled water, and drying the sediment overnight at 180°C.

References

1. Groot, W.J, Van der Lans, R. & Luyben, K. C. A. M. Technologies for butanol recovery integrated with fermentations. *Process. Biochem.* **27**, 61-75 (1992). doi:10.1016/0032-9592(92)80012-R
2. Qureshi, N. *et al.* Continuous solvent production by *Clostridium beijerinckii* BA101 immobilized by adsorption onto brick. *World. J. Microbiol. Biotechnol.* **16**, 377-382 (2000).
3. Huang, W.C., Ramey, D.E. & Yang, S.T. Continuous production of butanol by Clostridium acetobutylicum immobilized in a fibrous bed bioreactor. *Appl. Biochem. Biotechnol.* **115**, 887-898 (2004).
4. Chang, J. J. *et al.* Syntrophic coculture of aerobic Bacillus and anaerobic Clostridium for bio-fuels and bio-hydrogen production. *Int. J. Hydrogen. Energ.* **33**, 5137-5146 (2008).
5. Oshiro, M. *et al.* Efficient conversion of lactic acid to butanol with pH-stat continuous lactic acid and glucose feeding method by Clostridium saccharoperbutylacetonicum. *Appl. Microbiol. Biot.* 87, 1177-1185 (2010).
6. Lei, Y. *et al.* Fluorescence emission from CsI(Tl) crystal induced by high-energy carbon ions. *Opt. Mater.* **35**, 1179–1183 (2013).

7. Zhou, X. *et al.* Radiation induces acid tolerance of *Clostridium tyrobutyricum* and enhances bioproduction of butyric acid through a metabolic switch. *Biotechnol. Biofuels.* 7, 1 (2014).
8. Price, P. & McMillan, T.J. Use of the tetrazolium assay in measuring the response of human tumor cells to ionizing radiation. *Cancer. Res.* 50,1392–1396 (1990).
9. Buch, K. *et al.* Determination of cell survival after irradiation via clonogenic assay versus multiple MTT assay - a comparative study. *Radiat. Oncol.* 7, 1 (2012).
10. Harris, L. M. *et al.* Characterization of recombinant strains of the *Clostridium acetobutylicum* butyrate kinase inactivation mutant: need for new phenomenological models for solventogenesis and butanol inhibition?. *Biotechnol. Bioeng.* 67, 1-11 (2000).
11. Bogin, O. *et al.* Enhanced thermal stability of Clostridium beijerinckii alcohol dehydrogenase after strategic substitution of amino acid residues with prolines from the homologous thermophilic Thermoanaerobacter brockii alcohol dehydrogenase. *Protein. Science*. 7, 1156-1163 (1998).
12. Wucherpfennig, T., Hestler, T. & Krull, R. Morphology engineering–osmolality and its effect on *Aspergillus niger* morphology and productivity. *Microb. Cell. Fact.* 10, 58 (2011).
13. Yang, S. J. *et al.* Efficient degradation of lignocellulosic plant biomass, without pretreatment, by the thermophilic anaerobe "Anaerocellum thermophilum" DSM 6725. *Appl. Environ. Microbiol.* 74, 4762–4769 (2009).
14. Zhu, Y. & Yang, S.T. Adaptation of *Clostridium tyrobutyricum* for enhanced tolerance to butyric acid in a fibrous-bed bioreactor. *Biotechnol. Prog.* 19, 365–372 (2003).
