# Supplementary material for: Simplifying multidimensional fermentation dataset analysis and visualization: One step closer to capturing high-quality mutant strains
Source: Sci Rep. 2017 Jan 3;7:39875. doi: 10.1038/srep39875 (PMC5206668; doi:10.1038/srep39875)
Supplement: Supplementary Information 2 [file srep39875-s2.pdf]

Supplementary Information

Simplifying multidimensional fermentation dataset analysis and visualization: One step closer to capturing high-quality mutant strains

Xiang Zhou[1, †, *] , Dan Xu[1, †] and Ting-Ting Jiang[1,2,†]

Author affiliations

[1] Institute of Modern Physics, Chinese Academy of Sciences, 509 Nanchang Rd., Lanzhou, Gansu, P.R.China 730000

[2] University of Chinese Academy of Sciences, 19 A Yuquan Rd, Shijingshan District, Beijing, P.R.China 100049

†These authors contributed equally to this work.

Corresponding Author:

Xiang Zhou

***Corresponding Email address:** syannovich@gmail.com or syannovich@impcas.ac.cn

Phone: 86-931-4969688 Fax: +86-931-4969698

Supplementary Information: 13 pages, 1 table

Table S1. Mutant strain expression data sets with many variables: The effect of increasing the butyric acid concentration on the fermentation yield from the substrate, the butanol fermentation productivities of the 329 mutant strains supplemented with 5.0-g/L butyric acid, and the effect of butyric acid addition on the maximal specific growth rates for the 329 mutant strains (fermentation was carried out in serum bottles).

Serial number	*No. mutants*	*Butanol Productivity (g/L/h)*	*Butanol Yield (g/g)*	*Solvents (ABE) Yield (g/g)*	*Acetone Yield (g/g)*	*Ethanol Yield (g/g)*	*Maximal specific growth rate (added butyrate 5.0 g/L) μ_{max} (h^{-1})*	*Maximal specific growth rate (added butyrate 6.5 g/L) μ_{max} (h^{-1})*	*Maximal specific growth rate (added butyrate 8.5 g/L) μ_{max} (h^{-1})*
1.	FS-JWS-Z-34	0.062	0.325	0.417	0.121	0.051	0.217	0.162	0.096
2.	FS-JWS-Z-114	0.069	0.331	0.44	0.122	0.06	0.235	0.157	0.094
3.	FS-JWS-Z-1-34	0.07	0.349	0.458	0.127	0.06	0.233	0.162	0.098
4.	FS-JWS-D-3-124	0.066	0.36	0.46	0.138	0.059	0.226	0.156	0.091
5.	FS-JWS-Z-56	0.07	0.322	0.448	0.121	0.06	0.221	0.144	0.095
6.	FS-JWS-D-71	0.061	0.328	0.458	0.132	0.061	0.228	0.146	0.107
7.	FS-JWS-Z-71-36	0.066	0.33	0.458	0.128	0.059	0.234	0.156	0.093
8.	FS-JWS-B-78	0.069	0.34	0.448	0.123	0.059	0.224	0.155	0.09
9.	FS-JWS-Q-91-43	0.064	0.342	0.458	0.127	0.059	0.235	0.143	0.089
10.	FS-JWS-Q-14-8	0.065	0.36	0.454	0.123	0.059	0.226	0.144	0.096
11.	FS-JWS-Q-4-247	0.065	0.366	0.462	0.128	0.059	0.238	0.151	0.101
12.	FS-JWS-F-32	0.061	0.338	0.46	0.124	0.059	0.228	0.162	0.106
13.	FS-JWS-F-36-45	0.07	0.36	0.456	0.136	0.06	0.224	0.159	0.107
14.	FS-JWS-F-378-4	0.063	0.351	0.458	0.126	0.059	0.223	0.154	0.105
15.	FS-JWS-F-113-6	0.063	0.355	0.468	0.13	0.06	0.229	0.155	0.107
16.	FS-JWS-R-36	0.072	0.322	0.447	0.129	0.06	0.226	0.153	0.102
17.	FS-JWS-R-46-71	0.066	0.335	0.443	0.135	0.059	0.229	0.148	0.091

18.	**FS-JWS-R-89-34**	0.061	0.343	0.45	0.122	0.059	0.233	0.159	0.106
19.	**FS-JWS-R-61**	0.071	0.341	0.456	0.129	0.059	0.234	0.148	0.095
20.	**FS-JWS-R-61-23**	0.073	0.349	0.463	0.132	0.059	0.228	0.153	0.105
21.	**FS-JWS-T-72-69**	0.064	0.335	0.465	0.138	0.061	0.226	0.147	0.099
22.	**FS-JWS-T-35-245**	0.063	0.327	0.451	0.12	0.059	0.231	0.152	0.092
23.	**FS-JWS-T-145**	0.063	0.329	0.444	0.123	0.059	0.23	0.157	0.107
24.	**FS-JWS-T-3-134**	0.07	0.335	0.45	0.121	0.06	0.22	0.159	0.104
25.	**FS-JWS-T-312-11**	0.063	0.325	0.448	0.129	0.061	0.225	0.153	0.105
26.	**FS-JWS-T-312-71**	0.064	0.366	0.459	0.126	0.06	0.232	0.149	0.101
27.	**FS-JWS-T-312-22**	0.067	0.33	0.448	0.135	0.059	0.238	0.152	0.1
28.	**FS-JWS-T-346-2**	0.062	0.35	0.461	0.134	0.06	0.228	0.154	0.089
29.	**FS-JWS-T-346-24**	0.065	0.331	0.461	0.122	0.061	0.231	0.148	0.1
30.	**FS-JWS-PE-13**	0.067	0.36	0.466	0.134	0.061	0.23	0.146	0.092
31.	**FS-JWS-PE-24-34**	0.073	0.367	0.458	0.126	0.061	0.235	0.155	0.089
32.	**FS-JWS-PE-12-71**	0.062	0.322	0.449	0.123	0.06	0.238	0.154	0.092
33.	**FS-JWS-PE-3-171**	0.065	0.353	0.454	0.138	0.059	0.234	0.146	0.103
34.	**FS-JWS-PE-6-251**	0.064	0.337	0.44	0.122	0.06	0.232	0.146	0.093
35.	**FS-JWS-PE-19-67**	0.073	0.366	0.444	0.129	0.061	0.237	0.146	0.094
36.	**FS-JWS-DS-1545**	0.073	0.34	0.445	0.119	0.059	0.232	0.162	0.106
37.	**FS-JWS-DS-3527**	0.067	0.33	0.445	0.127	0.06	0.229	0.15	0.099
38.	**FS-JWS-DS-4271**	0.073	0.332	0.46	0.122	0.059	0.23	0.147	0.103
39.	**FS-JWS-DS-D-36**	0.073	0.333	0.466	0.132	0.06	0.22	0.154	0.105
40.	**FS-JWS-DS-D-70**	0.071	0.342	0.441	0.132	0.06	0.225	0.163	0.096
41.	**FS-JWS-DS-D-98**	0.069	0.326	0.464	0.136	0.059	0.234	0.15	0.103
42.	**FS-JWS-DS-F-3**	0.065	0.363	0.459	0.133	0.06	0.228	0.159	0.107
43.	**FS-JWS-DS-F-56**	0.069	0.328	0.459	0.129	0.059	0.227	0.158	0.089
44.	**FS-JWS-DS-F-261**	0.071	0.338	0.453	0.137	0.059	0.232	0.158	0.093
45.	**FS-JWS-DS-F-172**	0.069	0.361	0.449	0.138	0.06	0.237	0.159	0.089
46.	**FS-JWS-DS-F-296**	0.07	0.32	0.45	0.138	0.059	0.221	0.151	0.101

47.	**FS-JWS-DS-F-413**	0.064	0.332	0.441	0.136	0.061	0.221	0.149	0.099
48.	**FS-JWS-DS-F-527**	0.072	0.344	0.459	0.127	0.061	0.22	0.147	0.104
49.	**FS-JWS-HG-6-16**	0.065	0.344	0.468	0.126	0.061	0.227	0.157	0.09
50.	**FS-JWS-HG-9-27**	0.068	0.337	0.448	0.129	0.059	0.235	0.157	0.089
51.	**FS-JWS-HG-10-3**	0.07	0.362	0.454	0.126	0.059	0.23	0.154	0.089
52.	**FS-JWS-HG-10-9**	0.063	0.335	0.454	0.12	0.06	0.227	0.157	0.089
53.	**FS-JWS-HG-114**	0.072	0.34	0.468	0.135	0.059	0.226	0.161	0.103
54.	**FS-JWS-HG-145**	0.069	0.366	0.458	0.125	0.059	0.236	0.157	0.096
55.	**FS-JWS-HG-1632**	0.065	0.358	0.445	0.133	0.061	0.225	0.15	0.092
56.	**FS-JWS-HG-1564**	0.064	0.34	0.441	0.133	0.06	0.221	0.162	0.104
57.	**FS-JWS-HG-2357**	0.072	0.356	0.463	0.134	0.06	0.232	0.144	0.103
58.	**FS-JWS-AF-263**	0.065	0.333	0.44	0.128	0.061	0.232	0.147	0.104
59.	**FS-JWS-AF-397**	0.066	0.356	0.44	0.123	0.061	0.227	0.159	0.094
60.	**FS-JWS-AF-634**	0.067	0.319	0.451	0.125	0.06	0.227	0.157	0.101
61.	**FS-JWS-AF-967**	0.062	0.33	0.45	0.134	0.059	0.222	0.152	0.098
62.	**FS-JWS-AF-1172**	0.061	0.361	0.449	0.135	0.059	0.219	0.158	0.101
63.	**FS-JWS-AF-1283**	0.064	0.347	0.462	0.135	0.06	0.224	0.143	0.102
64.	**FS-JWS-AF-2468**	0.066	0.333	0.454	0.12	0.059	0.229	0.155	0.106
65.	**FS-JWS-AF-F-23**	0.068	0.325	0.451	0.129	0.059	0.222	0.149	0.108
66.	**FS-JWS-AF-F-43**	0.065	0.336	0.46	0.119	0.06	0.22	0.163	0.097
67.	**FS-JWS-AF-F-246**	0.073	0.354	0.452	0.132	0.06	0.233	0.148	0.095
68.	**FS-JWS-AF-F-356**	0.071	0.36	0.448	0.137	0.059	0.233	0.143	0.097
69.	**FS-JWS-AF-F-687**	0.071	0.329	0.467	0.137	0.06	0.228	0.157	0.106
70.	**FS-JWS-AF-F-956**	0.07	0.359	0.46	0.127	0.061	0.224	0.158	0.099
71.	**FS-JWS-VJ-24-5**	0.067	0.356	0.451	0.134	0.059	0.235	0.157	0.095
72.	**FS-JWS-VJ-24-72**	0.071	0.331	0.464	0.122	0.06	0.233	0.143	0.094
73.	**FS-JWS-VJ-24-89**	0.064	0.333	0.447	0.133	0.061	0.236	0.151	0.107
74.	**FS-JWS-VJ-T-8**	0.062	0.342	0.441	0.13	0.06	0.235	0.144	0.091
75.	**FS-JWS-VJ-T-56**	0.065	0.35	0.453	0.131	0.059	0.236	0.154	0.093

76.	**FS-JWS-VJ-T-97**	0.072	0.319	0.449	0.133	0.06	0.227	0.154	0.103
77.	**FS-JWS-VJ-T-183**	0.064	0.332	0.445	0.134	0.059	0.221	0.155	0.103
78.	**FS-JWS-VJ-T-346**	0.061	0.351	0.448	0.121	0.061	0.234	0.145	0.1
79.	**FS-JWS-VJ-W-32**	0.067	0.35	0.447	0.126	0.06	0.219	0.156	0.108
80.	**FS-JWS-VJ-W-78**	0.066	0.328	0.468	0.12	0.061	0.235	0.143	0.09
81.	**FS-JWS-VJ-W-89**	0.068	0.341	0.467	0.133	0.059	0.227	0.148	0.095
82.	**FS-JWS-VJ-W-12**	0.072	0.35	0.466	0.135	0.06	0.236	0.153	0.103
83.	**FS-JWS-VJ-W-17**	0.065	0.365	0.441	0.137	0.06	0.236	0.162	0.098
84.	**FS-JWS-Q-1-368**	0.061	0.345	0.455	0.12	0.061	0.227	0.157	0.097
85.	**FS-JWS-Q-1-765**	0.066	0.36	0.45	0.131	0.061	0.23	0.161	0.107
86.	**FS-JWS-Q-1-963**	0.068	0.319	0.443	0.125	0.061	0.226	0.147	0.106
87.	**FS-JWS-Q-26-6**	0.068	0.354	0.468	0.129	0.06	0.233	0.161	0.107
88.	**FS-JWS-Q-26-26**	0.061	0.321	0.443	0.137	0.06	0.231	0.153	0.092
89.	**FS-JWS-QV-98-6**	0.073	0.352	0.454	0.133	0.059	0.219	0.144	0.089
90.	**FS-JWS-QV-98-9**	0.07	0.368	0.453	0.137	0.059	0.222	0.162	0.094
91.	**FS-JWS-QV-G8-6**	0.073	0.328	0.456	0.128	0.061	0.222	0.163	0.094
92.	**FS-JWS-QV-G8-4**	0.068	0.337	0.459	0.127	0.061	0.228	0.154	0.092
93.	**FS-JWS-QV-J4-9**	0.067	0.34	0.453	0.123	0.06	0.231	0.156	0.1
94.	**FS-JWS-QV-J5-63**	0.062	0.339	0.443	0.13	0.061	0.22	0.156	0.107
95.	**FS-JWS-QV-J30-3**	0.068	0.334	0.44	0.125	0.059	0.235	0.158	0.09
96.	**FS-JWS-QV-J30-9**	0.063	0.334	0.444	0.127	0.06	0.236	0.153	0.092
97.	**FS-JWS-QV-J94-3**	0.072	0.335	0.464	0.129	0.06	0.237	0.151	0.108
98.	**FS-JWS-QV-J94-8**	0.071	0.345	0.454	0.138	0.059	0.231	0.156	0.094
99.	**FS-JWS-QV-M3-9**	0.071	0.36	0.459	0.127	0.06	0.236	0.15	0.097
100.	**FS-JWS-QV-M9-2**	0.069	0.358	0.459	0.121	0.059	0.227	0.154	0.096
101.	**FS-JWS-QV-M12**	0.073	0.362	0.497	0.121	0.061	0.224	0.143	0.103
102.	**FS-JWS-QV-M35**	0.07	0.39	0.495	0.115	0.059	0.228	0.151	0.09
103.	**FS-JWS-SN-6-63**	0.069	0.4	0.49	0.114	0.06	0.231	0.163	0.101
104.	**FS-JWS-SN-6-97**	0.064	0.349	0.481	0.121	0.06	0.219	0.156	0.104

105.	**FS-JWS-SN-6-A2**	0.066	0.389	0.489	0.119	0.059	0.222	0.162	0.092
106.	**FS-JWS-SN-6-A9**	0.07	0.404	0.49	0.127	0.06	0.232	0.144	0.105
107.	**FS-JWS-SN-25-2**	0.065	0.383	0.488	0.111	0.06	0.225	0.156	0.099
108.	**FS-JWS-SN-25-9**	0.068	0.388	0.485	0.116	0.061	0.225	0.143	0.095
109.	**FS-JWS-SN-79-6**	0.068	0.418	0.494	0.113	0.06	0.224	0.146	0.093
110.	**FS-JWS-SN-84-6**	0.064	0.398	0.489	0.13	0.061	0.224	0.153	0.096
111.	**FS-JWS-SN-1D-4**	0.068	0.373	0.489	0.126	0.06	0.226	0.147	0.1
112.	**FS-JWS-SN-9D-6**	0.073	0.382	0.487	0.118	0.061	0.228	0.161	0.09
113.	**FS-JWS-SN-9F-3**	0.072	0.408	0.485	0.126	0.06	0.234	0.143	0.09
114.	**FS-JWS-SN-9F-7**	0.064	0.365	0.491	0.109	0.06	0.222	0.153	0.092
115.	**FS-JWS-SN-9F-9**	0.071	0.364	0.491	0.111	0.06	0.228	0.161	0.108
116.	**FS-TU-3R-2**	0.066	0.416	0.486	0.116	0.061	0.23	0.146	0.101
117.	**FS-TU-3R-9**	0.067	0.39	0.485	0.123	0.06	0.219	0.156	0.095
118.	**FS-TU-3R-17**	0.063	0.377	0.483	0.132	0.061	0.231	0.159	0.102
119.	**FS-TU-3R-17A-6**	0.062	0.354	0.495	0.127	0.06	0.238	0.15	0.093
120.	**FS-TU-3R-17A-24**	0.066	0.36	0.481	0.12	0.06	0.224	0.154	0.103
121.	**FS-TU-3R-17A-39**	0.067	0.339	0.487	0.113	0.061	0.247	0.147	0.106
122.	**FS-TU-3R-34H-3**	0.067	0.378	0.489	0.121	0.061	0.246	0.159	0.092
123.	**FS-TU-3R-34H-6**	0.062	0.364	0.486	0.124	0.06	0.245	0.16	0.092
124.	**FS-TU-131R-1-5**	0.062	0.412	0.485	0.113	0.061	0.24	0.147	0.104
125.	**FS-TU-131R-1-9**	0.072	0.353	0.482	0.114	0.061	0.243	0.15	0.097
126.	**FS-TU-131R-1-67**	0.067	0.402	0.487	0.12	0.059	0.244	0.161	0.1
127.	**FS-TU-131R-1-83**	0.068	0.386	0.485	0.128	0.061	0.244	0.156	0.097
128.	**FS-TU-E1R-3-543**	0.07	0.347	0.486	0.129	0.061	0.246	0.153	0.095
129.	**FS-TU-E1R-3-963**	0.07	0.396	0.484	0.122	0.06	0.247	0.145	0.106
130.	**FS-TU-E1R-9-A3**	0.062	0.399	0.482	0.122	0.059	0.239	0.15	0.094
131.	**FS-TU-E1R-9-A42**	0.066	0.407	0.49	0.119	0.061	0.245	0.161	0.106
132.	**FS-TU-E1R-9-B36**	0.063	0.386	0.49	0.123	0.061	0.247	0.144	0.105
133.	**FS-TU-E1R-9-B52**	0.063	0.405	0.486	0.127	0.061	0.24	0.143	0.098

134.	**FS-TU-E1R-9-B-2**	0.061	0.391	0.484	0.126	0.061	0.242	0.155	0.108
135.	**FS-TU-E1R-9-B-9**	0.062	0.395	0.493	0.123	0.061	0.242	0.155	0.089
136.	**FS-TU-EA-3-8**	0.062	0.402	0.493	0.131	0.061	0.24	0.15	0.106
137.	**FS-TU-EA-3-27**	0.066	0.387	0.481	0.116	0.087	0.243	0.155	0.091
138.	**FS-TU-EA-3-136**	0.068	0.39	0.489	0.129	0.091	0.24	0.16	0.097
139.	**FS-TU-EA-3-247**	0.073	0.409	0.487	0.125	0.088	0.241	0.151	0.107
140.	**FS-TU-EA-3-681**	0.062	0.366	0.491	0.111	0.08	0.242	0.153	0.099
141.	**FS-TU-EA-3-762**	0.066	0.389	0.492	0.119	0.103	0.242	0.158	0.093
142.	**FS-TU-EA-3-1436**	0.069	0.339	0.493	0.113	0.11	0.241	0.153	0.1
143.	**FS-TU-EA-W-14**	0.07	0.416	0.485	0.13	0.091	0.243	0.155	0.092
144.	**FS-TU-EA-W-29**	0.071	0.346	0.486	0.132	0.092	0.24	0.158	0.094
145.	**FS-TU-EA-W-36**	0.068	0.392	0.482	0.131	0.083	0.246	0.156	0.092
146.	**FS-TU-EA-W-131**	0.063	0.352	0.483	0.122	0.111	0.246	0.154	0.092
147.	**FS-TU-EA-W-223**	0.071	0.364	0.483	0.118	0.105	0.242	0.146	0.101
148.	**FS-TU-EA-W-354**	0.072	0.396	0.49	0.115	0.109	0.24	0.154	0.097
149.	**FS-TU-EA-80-2**	0.066	0.382	0.482	0.128	0.112	0.243	0.156	0.089
150.	**FS-TU-EA-80-29**	0.065	0.381	0.492	0.126	0.081	0.241	0.153	0.096
151.	**FS-TU-EA-80-31**	0.067	0.355	0.483	0.13	0.112	0.245	0.148	0.102
152.	**FS-TU-EA-80-123**	0.063	0.404	0.492	0.118	0.088	0.243	0.148	0.095
153.	**FS-TU-EA-AX-12**	0.068	0.417	0.492	0.128	0.113	0.246	0.153	0.089
154.	**FS-TU-EA-AX-19**	0.071	0.394	0.482	0.13	0.128	0.24	0.162	0.1
155.	**FS-TU-EA-AX-35**	0.073	0.408	0.482	0.113	0.109	0.244	0.153	0.09
156.	**FS-TU-EA-AX-71**	0.063	0.411	0.489	0.124	0.13	0.242	0.143	0.099
157.	**FS-TU-EA-AJ-12**	0.061	0.396	0.493	0.118	0.084	0.243	0.155	0.1
158.	**FS-TU-EA-AJ-53**	0.068	0.403	0.487	0.117	0.127	0.242	0.148	0.092
159.	**FS-TU-EA-AJ-97**	0.069	0.358	0.49	0.126	0.09	0.24	0.152	0.1
160.	**FS-TU-EA-AJ-264**	0.071	0.344	0.49	0.111	0.101	0.239	0.161	0.091
161.	**FS-TU-EA-AJ-720**	0.073	0.406	0.481	0.119	0.099	0.244	0.148	0.095
162.	**FS-TU-EA-AJ-943**	0.07	0.408	0.485	0.121	0.117	0.246	0.151	0.091

163.	FS-TU-EA-FG-39	0.063	0.345	0.484	0.126	0.123	0.246	0.145	0.097
164.	FS-TU-EA-FG-78	0.063	0.364	0.492	0.122	0.125	0.24	0.145	0.108
165.	FS-TU-EA-FG-138	0.07	0.379	0.489	0.119	0.079	0.241	0.153	0.108
166.	FS-TU-EA-FG-251	0.062	0.361	0.494	0.12	0.115	0.241	0.149	0.105
167.	FS-TU-EA-FG-364	0.067	0.405	0.481	0.123	0.092	0.247	0.145	0.091
168.	FS-TU-ETQ-4A-4	0.064	0.391	0.497	0.115	0.085	0.247	0.151	0.102
169.	FS-TU-ETQ-4A-9	0.065	0.376	0.484	0.12	0.08	0.239	0.147	0.103
170.	FS-TU-ETQ-9A-3	0.065	0.348	0.494	0.127	0.125	0.242	0.151	0.105
171.	FS-TU-ETQ-9A-5	0.071	0.393	0.486	0.122	0.106	0.244	0.145	0.097
172.	FS-TU-ETQ-129B	0.069	0.353	0.488	0.114	0.117	0.244	0.152	0.091
173.	FS-TU-ETQ-247B	0.073	0.405	0.496	0.109	0.084	0.243	0.163	0.104
174.	FS-TU-ETQ-517B	0.069	0.41	0.485	0.115	0.092	0.24	0.159	0.1
175.	FS-TU-ETQ-DM17	0.071	0.399	0.484	0.111	0.095	0.244	0.151	0.108
176.	FS-TU-ETQ-DM52	0.064	0.369	0.49	0.118	0.129	0.243	0.162	0.092
177.	FS-TU-ETQ-DM72	0.072	0.375	0.484	0.123	0.124	0.245	0.143	0.107
178.	FS-TU-ETQ-DM93	0.068	0.341	0.496	0.111	0.102	0.247	0.158	0.104
179.	FS-TU-ETQ-M-23	0.067	0.387	0.481	0.118	0.102	0.24	0.161	0.095
180.	FS-TU-ETQ-M-37	0.063	0.392	0.494	0.121	0.092	0.246	0.145	0.099
181.	FS-TU-ETQ-M-54	0.061	0.381	0.496	0.111	0.126	0.239	0.161	0.095
182.	FS-TU-ETQ-M-67	0.062	0.415	0.487	0.11	0.099	0.244	0.155	0.111
183.	FS-TU-ETQ-M1-6	0.067	0.405	0.487	0.121	0.091	0.242	0.148	0.11
184.	FS-TU-ETQ-M1-9	0.072	0.348	0.492	0.126	0.121	0.242	0.155	0.102
185.	FS-TU-ETQ-M10	0.063	0.361	0.491	0.11	0.119	0.247	0.163	0.1
186.	FS-TU-ETQ-M167	0.068	0.379	0.49	0.128	0.102	0.242	0.162	0.104
187.	FS-TU-ETQ-M351	0.061	0.361	0.487	0.11	0.105	0.239	0.152	0.109
188.	FS-TU-ETQ-M672	0.067	0.384	0.489	0.109	0.111	0.24	0.153	0.1
189.	FS-TU-ETQ-MO6	0.072	0.354	0.494	0.109	0.097	0.243	0.16	0.109
190.	FS-TU-ETQ-MO9	0.068	0.386	0.483	0.119	0.1	0.241	0.149	0.112
191.	FS-TU-ETQ-MO1	0.073	0.388	0.484	0.124	0.122	0.241	0.15	0.1

192.	**FS-TU-ETQ-MU7**	0.07	0.405	0.495	0.11	0.081	0.247	0.146	0.107
193.	**FS-TU-ETQ-MU9**	0.071	0.351	0.488	0.127	0.118	0.24	0.162	0.11
194.	**FS-SXT-A9-23**	0.07	0.361	0.483	0.119	0.099	0.243	0.162	0.106
195.	**FS-SXT-A9-56**	0.069	0.376	0.487	0.129	0.097	0.246	0.146	0.113
196.	**FS-SXT-A9-71**	0.068	0.378	0.489	0.13	0.087	0.246	0.151	0.101
197.	**FS-SXT-A9-347**	0.065	0.346	0.488	0.11	0.122	0.242	0.143	0.11
198.	**FS-SXT-A9-1126**	0.063	0.361	0.483	0.121	0.117	0.244	0.152	0.106
199.	**FS-SXT-A9-1492**	0.067	0.381	0.496	0.115	0.101	0.239	0.151	0.101
200.	**FS-SXT-AP-R-61**	0.069	0.379	0.492	0.129	0.104	0.239	0.158	0.113
201.	**FS-SXT-AP-R-83**	0.066	0.438	0.5	0.124	0.129	0.246	0.158	0.106
202.	**FS-SXT-AP-R-226**	0.071	0.438	0.49	0.111	0.126	0.239	0.158	0.101
203.	**FS-SXT-AP-R-641**	0.07	0.432	0.501	0.127	0.109	0.247	0.148	0.113
204.	**FS-SXT-AP-R3-12**	0.072	0.421	0.481	0.115	0.13	0.242	0.16	0.105
205.	**FS-SXT-AP-R3-76**	0.063	0.421	0.505	0.128	0.09	0.24	0.16	0.113
206.	**FS-SXT-AP-R72-3**	0.071	0.415	0.517	0.123	0.121	0.245	0.162	0.111
207.	**FS-SXT-AP-R72-9**	0.067	0.438	0.52	0.128	0.089	0.24	0.163	0.104
208.	**FS-SXT-AP-R119**	0.07	0.406	0.485	0.129	0.08	0.247	0.143	0.108
209.	**FS-SXT-AP-R183**	0.065	0.418	0.52	0.114	0.102	0.244	0.148	0.106
210.	**FS-SXT-AP-R467**	0.072	0.403	0.52	0.13	0.09	0.245	0.145	0.107
211.	**FS-SXT-AP-R631**	0.07	0.401	0.528	0.117	0.126	0.247	0.159	0.108
212.	**FS-SXT-LE-B-65**	0.068	0.408	0.533	0.127	0.082	0.243	0.151	0.109
213.	**FS-SXT-LE-B-89**	0.07	0.432	0.511	0.125	0.119	0.242	0.143	0.099
214.	**FS-SXT-LE-B-113**	0.069	0.426	0.484	0.125	0.083	0.239	0.162	0.106
215.	**FS-SXT-LE-B-572**	0.072	0.411	0.492	0.121	0.09	0.244	0.158	0.1
216.	**FS-SXT-LE-BH-52**	0.067	0.404	0.529	0.115	0.124	0.242	0.149	0.106
217.	**FS-SXT-LE-BH-73**	0.061	0.435	0.537	0.12	0.089	0.243	0.148	0.105
218.	**FS-SXT-LE-BH-85**	0.062	0.408	0.535	0.121	0.11	0.246	0.148	0.106
219.	**FS-SXT-LE-BH-91**	0.062	0.4	0.52	0.128	0.123	0.239	0.157	0.113
220.	**FS-SXT-LE-BH-A1**	0.066	0.433	0.531	0.119	0.081	0.246	0.148	0.112

221.	**FS-SXT-LE-BH-A7**	0.073	0.405	0.53	0.118	0.128	0.24	0.157	0.111
222.	**FS-SXT-LE-BH-P9**	0.066	0.428	0.504	0.111	0.062	0.241	0.151	0.106
223.	**FS-SXT-QDV-22**	0.071	0.417	0.517	0.125	0.06	0.245	0.152	0.108
224.	**FS-SXT-QDV-32**	0.068	0.432	0.49	0.118	0.057	0.244	0.156	0.11
225.	**FS-SXT-QDV-371**	0.064	0.418	0.509	0.132	0.068	0.239	0.158	0.099
226.	**FS-SXT-QDV-456**	0.063	0.4	0.486	0.114	0.067	0.247	0.149	0.107
227.	**FS-SXT-QDV-863**	0.073	0.431	0.504	0.117	0.066	0.244	0.155	0.111
228.	**FS-SXT-QDV-923**	0.069	0.425	0.503	0.11	0.061	0.245	0.163	0.104
229.	**FS-SXT-QDV-A6**	0.061	0.41	0.496	0.118	0.056	0.244	0.149	0.109
230.	**FS-SXT-QDV-A9**	0.072	0.415	0.517	0.125	0.068	0.247	0.163	0.109
231.	**FS-SXT-QDV-A11**	0.073	0.435	0.522	0.114	0.058	0.247	0.145	0.11
232.	**FS-SXT-QDV-A19**	0.063	0.4	0.517	0.125	0.056	0.245	0.147	0.104
233.	**FS-SXT-QDV-U1**	0.068	0.418	0.527	0.121	0.063	0.246	0.15	0.099
234.	**FS-SXT-QDV-U3**	0.061	0.412	0.524	0.129	0.057	0.241	0.147	0.112
235.	**FS-SXT-QDV-U27**	0.066	0.411	0.532	0.124	0.062	0.247	0.152	0.11
236.	**FS-SXT-QDV-VA2**	0.069	0.423	0.485	0.127	0.066	0.247	0.147	0.102
237.	**FS-SXT-QDV-VA9**	0.072	0.399	0.501	0.121	0.065	0.247	0.151	0.103
238.	**FS-SXT-QDV-VJ7**	0.073	0.431	0.518	0.119	0.056	0.24	0.146	0.108
239.	**FS-SXT-QDV-VJ9**	0.062	0.394	0.496	0.105	0.047	0.227	0.154	0.102
240.	**FS-SXT-GC-V-3**	0.072	0.423	0.521	0.123	0.09	0.246	0.15	0.104
241.	**FS-SXT-GC-V-34**	0.062	0.421	0.487	0.119	0.096	0.242	0.153	0.101
242.	**FS-SXT-GC-V-56**	0.062	0.438	0.491	0.119	0.107	0.244	0.157	0.112
243.	**FS-SXT-GC-V-127**	0.062	0.427	0.503	0.132	0.069	0.239	0.158	0.099
244.	**FS-SXT-GC-V-243**	0.063	0.42	0.48	0.111	0.081	0.246	0.152	0.102
245.	**FS-SXT-GC-V-363**	0.062	0.415	0.5	0.117	0.09	0.243	0.157	0.108
246.	**FS-SXT-GC-V7-24**	0.073	0.421	0.517	0.117	0.095	0.239	0.158	0.099
247.	**FS-SXT-GC-V7-43**	0.067	0.429	0.499	0.111	0.069	0.241	0.148	0.11
248.	**FS-SXT-GC-V9-13**	0.064	0.434	0.531	0.121	0.101	0.239	0.144	0.109
249.	**FS-SXT-GC-V9-26**	0.067	0.41	0.52	0.12	0.08	0.239	0.148	0.111

250.	**FS-SXT-GC-V9-34**	0.067	0.404	0.51	0.122	0.12	0.242	0.148	0.106
251.	**FS-SXT-GC-V9-46**	0.067	0.438	0.494	0.119	0.076	0.247	0.156	0.111
252.	**FS-SXT-GC-V9-77**	0.068	0.435	0.533	0.113	0.117	0.243	0.157	0.11
253.	**FS-SXT-GC-V9-81**	0.061	0.42	0.48	0.116	0.1	0.245	0.153	0.11
254.	**FS-SXT-GC-V9-93**	0.062	0.4	0.524	0.132	0.114	0.247	0.156	0.113
255.	**FS-HYR-T-63**	0.073	0.42	0.489	0.129	0.103	0.242	0.152	0.103
256.	**FS-HYR-T-97**	0.07	0.435	0.481	0.113	0.083	0.247	0.155	0.106
257.	**FS-ZKS-TF-A12-9**	0.062	0.42	0.514	0.121	0.1	0.239	0.144	0.111
258.	**FS- ZKS-TF-A35-7**	0.073	0.418	0.494	0.109	0.1	0.245	0.156	0.103
259.	**FS-ZKS-TF-A41-3**	0.064	0.41	0.517	0.113	0.081	0.241	0.151	0.109
260.	**FS-ZKS-TF-A46-5**	0.062	0.416	0.489	0.12	0.102	0.242	0.162	0.099
261.	**FS-ZKS-TF-A51-1**	0.068	0.404	0.499	0.111	0.105	0.241	0.154	0.113
262.	**FS-ZKS-TF-AT1-6**	0.064	0.428	0.532	0.131	0.084	0.243	0.145	0.113
263.	**FS-ZKS-TF-AT3-9**	0.062	0.406	0.537	0.129	0.09	0.246	0.143	0.11
264.	**FS-ZKS-TF-AT6-5**	0.065	0.416	0.534	0.11	0.088	0.245	0.155	0.103
265.	**FS-ZKS -TF-AT8-3**	0.062	0.403	0.532	0.131	0.081	0.243	0.157	0.107
266.	**FS-ZKS-TF-AT9-9**	0.063	0.419	0.518	0.121	0.069	0.245	0.157	0.107
267.	**FS-ZKS-TF-DT-74**	0.068	0.423	0.513	0.122	0.113	0.24	0.16	0.106
268.	**FS-ZKS-TF-DT-81**	0.073	0.436	0.479	0.121	0.093	0.24	0.144	0.107
269.	**FS-ZKS-TF-DT-92**	0.066	0.408	0.524	0.11	0.084	0.24	0.149	0.106
270.	**FS-ZKS-TF-MT18**	0.072	0.435	0.524	0.113	0.091	0.241	0.152	0.113
271.	**FS-ZKS-TF-MT53**	0.07	0.403	0.526	0.12	0.095	0.247	0.144	0.103
272.	**FS-ZKS-TF-MT67**	0.072	0.429	0.48	0.118	0.11	0.245	0.153	0.113
273.	**FS-ZKS-TF-MT74**	0.067	0.429	0.53	0.123	0.091	0.24	0.15	0.106
274.	**FS-ZKS-TF-MT83**	0.073	0.427	0.497	0.124	0.086	0.24	0.155	0.106
275.	**FS-ZKS-TF-MT96**	0.073	0.437	0.522	0.124	0.114	0.246	0.145	0.099
276.	**FS-ZKS-TF-XT-4**	0.063	0.409	0.523	0.112	0.081	0.241	0.158	0.104
277.	**FS-ZKS-TF-XT-9**	0.073	0.432	0.485	0.124	0.11	0.243	0.152	0.099
278.	**FS-ZKS-TF-XT-13**	0.067	0.427	0.49	0.12	0.082	0.243	0.151	0.109

279.	FS-ZKS-TF-XT-35	0.069	0.402	0.494	0.126	0.071	0.242	0.155	0.101
280.	FS-ZKS-TF-XT-61	0.07	0.401	0.523	0.116	0.069	0.241	0.151	0.107
281.	FS-ZKS-TF-XT-97	0.062	0.4	0.498	0.117	0.08	0.24	0.159	0.106
282.	FS-ZKS-TF-ZT1-4	0.068	0.431	0.492	0.114	0.114	0.242	0.148	0.103
283.	FS-ZKS-TF-ZT3-6	0.066	0.414	0.526	0.128	0.108	0.245	0.157	0.104
284.	FS-ZKS-TF-ZT7-9	0.073	0.406	0.512	0.117	0.073	0.242	0.163	0.104
285.	FS-ZKS-TF-ZT11	0.062	0.42	0.497	0.122	0.102	0.246	0.155	0.108
286.	FS-ZKS-TF-ZT34	0.073	0.431	0.491	0.126	0.069	0.242	0.147	0.11
287.	FS-ZKS-TF-ZT51	0.068	0.407	0.501	0.12	0.106	0.246	0.16	0.099
288.	FS-ZKS-TF-ZT83	0.071	0.427	0.485	0.128	0.074	0.243	0.155	0.1
289.	FS-ZKS-TF-ZT137	0.061	0.421	0.525	0.117	0.079	0.239	0.163	0.111
290.	FS-ZKS-TF-ZT383	0.073	0.424	0.534	0.125	0.102	0.239	0.147	0.112
291.	FS-ZKS -TF-ZT637	0.067	0.426	0.532	0.109	0.101	0.245	0.147	0.112
292.	FS-ZKS-TF-ZT835	0.067	0.411	0.525	0.11	0.069	0.239	0.158	0.111
293.	FS-GCJ-A-138	0.064	0.397	0.489	0.115	0.075	0.239	0.148	0.109
294.	FS-GCJ-A-361	0.07	0.406	0.516	0.125	0.11	0.246	0.151	0.112
295.	FS-GCJ-A-714	0.068	0.428	0.492	0.112	0.094	0.242	0.159	0.105
296.	FS-GCJ-A-837	0.063	0.41	0.497	0.114	0.072	0.243	0.145	0.104
297.	FS-GCJ-A-1352	0.067	0.426	0.49	0.123	0.094	0.24	0.158	0.102
298.	FS-GCJ-A-3652	0.065	0.428	0.508	0.118	0.096	0.243	0.148	0.107
299.	FS-GCJ-A79-343	0.066	0.421	0.523	0.132	0.074	0.241	0.152	0.1
300.	FS-GCJ-A79-642	0.063	0.418	0.502	0.118	0.112	0.246	0.152	0.109
301.	FS-GCJ-A234-36	0.064	0.426	0.517	0.124	0.09	0.239	0.159	0.1
302.	FS-GCJ-A234-237	0.072	0.413	0.505	0.126	0.096	0.241	0.156	0.107
303.	FS-GCJ-A234-364	0.073	0.418	0.511	0.119	0.069	0.239	0.151	0.112
304.	FS-GCJ-A234-439	0.073	0.428	0.485	0.127	0.082	0.239	0.147	0.1
305.	FS-GCJ-AS23-3A	0.068	0.403	0.537	0.123	0.072	0.24	0.148	0.103
306.	FS-GCJ-AS23-9A	0.071	0.402	0.526	0.111	0.102	0.245	0.144	0.11
307.	FS-GCJ-AS23-63R	0.07	0.402	0.527	0.114	0.116	0.239	0.155	0.101

308.	**FS-GCJ-AS91-95R**	0.062	0.407	0.481	0.125	0.093	0.245	0.154	0.105
309.	**FS-GCJ-AS139-1**	0.071	0.402	0.498	0.117	0.081	0.242	0.158	0.107
310.	**FS-GCJ-AS139-23**	0.073	0.422	0.519	0.123	0.072	0.243	0.153	0.1
311.	**FS-GCJ-AS139-36**	0.068	0.412	0.494	0.129	0.085	0.241	0.152	0.113
312.	**FS-GCJ-AS139-71**	0.072	0.428	0.533	0.127	0.089	0.247	0.159	0.108
313.	**FS-GCJ-AS139-86**	0.064	0.408	0.535	0.129	0.113	0.246	0.157	0.099
314.	**FS-GCJ-AS139-93**	0.069	0.417	0.53	0.121	0.087	0.243	0.15	0.111
315.	**FS-GCJ-FL-167**	0.068	0.404	0.505	0.114	0.071	0.246	0.153	0.108
316.	**FS-GCJ-FL-356**	0.073	0.431	0.529	0.112	0.078	0.241	0.161	0.113
317.	**FS-GCJ-FL-763**	0.066	0.406	0.508	0.114	0.089	0.239	0.158	0.106
318.	**FS-GCJ-FL-1361**	0.064	0.423	0.532	0.117	0.12	0.243	0.15	0.102
319.	**FS-GCJ-FL-2473**	0.062	0.406	0.521	0.125	0.089	0.241	0.163	0.106
320.	**FS-GCJ-FL-VB-24**	0.066	0.426	0.524	0.13	0.08	0.24	0.161	0.103
321.	**FS-GCJ-FL-VB-76**	0.07	0.416	0.49	0.124	0.118	0.243	0.159	0.111
322.	**FS-GCJ-FL-VB-81**	0.065	0.408	0.529	0.123	0.106	0.245	0.146	0.101
323.	**FS-GCJ-FL-VB-97**	0.064	0.4	0.515	0.129	0.105	0.244	0.15	0.107
324.	**FS-GCJ-FL-QS-13**	0.061	0.411	0.505	0.131	0.079	0.247	0.149	0.105
325.	**FS-GCJ-FL-QS-62**	0.064	0.399	0.481	0.122	0.074	0.246	0.162	0.11
326.	**FS-GCJ-FL-QS-73**	0.062	0.422	0.503	0.131	0.121	0.239	0.153	0.1
327.	**FS-GCJ-FL-QS-Y3**	0.07	0.397	0.513	0.109	0.07	0.24	0.156	0.099
328.	**FS-GCJ-FL-QS-Y5**	0.068	0.408	0.526	0.111	0.082	0.242	0.162	0.105
329.	**FS-GCJ-FL-QS-Y9**	0.061	0.414	0.504	0.114	0.079	0.236	0.151	0.097

*All the experiments were repeated 3 times and the average value was taken.
